# Altered resting-state functional connectivity in newborns with hypoxic ischemic encephalopathy assessed using high-density functional near-infrared spectroscopy

**DOI:** 10.1038/s41598-024-53256-0

**Published:** 2024-02-07

**Authors:** Lingkai Tang, Lilian M. N. Kebaya, Talal Altamimi, Alexandra Kowalczyk, Melab Musabi, Sriya Roychaudhuri, Homa Vahidi, Paige Meyerink, Sandrine de Ribaupierre, Soume Bhattacharya, Leandro Tristao Abi Ramia de Moraes, Keith St. Lawrence, Emma G. Duerden

**Affiliations:** 1 Biomedical Engineering, Faculty of Engineering, Western University, London, ON Canada; 2 Neuroscience, Schulich Faculty of Medicine and Dentistry, Western University, London, ON Canada; 3 Neonatal-Perinatal Medicine, Schulich Faculty of Medicine and Dentistry, Western University, London, ON Canada; 4 Department of Paediatrics, Division of Neonatal-Perinatal Medicine, Temerty Faculty of Medicine, University of Toronto, Toronto, ON Canada; 5 Clinical Neurological Sciences, Schulich Faculty of Medicine and Dentistry, Western University, London, ON Canada; 6 Medical Imaging, London Health Sciences Centre, Western University, London, ON Canada; 7 Medical Biophysics, Schulich Faculty of Medicine and Dentistry, Western University, London, ON Canada; 8 Applied Psychology, Faculty of Education, Western University, 1137 Western Rd, London, ON N6G 1G7 Canada

**Keywords:** Hypoxic-ischaemic encephalopathy, Paediatric research

## Abstract

Hypoxic-ischemic encephalopathy (HIE) results from a lack of oxygen to the brain during the perinatal period. HIE can lead to mortality and various acute and long-term morbidities. Improved bedside monitoring methods are needed to identify biomarkers of brain health. Functional near-infrared spectroscopy (fNIRS) can assess resting-state functional connectivity (RSFC) at the bedside. We acquired resting-state fNIRS data from 21 neonates with HIE (postmenstrual age [PMA] = 39.96), in 19 neonates the scans were acquired post-therapeutic hypothermia (TH), and from 20 term-born healthy newborns (PMA = 39.93). Twelve HIE neonates also underwent resting-state functional magnetic resonance imaging (fMRI) post-TH. RSFC was calculated as correlation coefficients amongst the time courses for fNIRS and fMRI data, respectively. The fNIRS and fMRI RSFC maps were comparable. RSFC patterns were then measured with graph theory metrics and compared between HIE infants and healthy controls. HIE newborns showed significantly increased clustering coefficients, network efficiency and modularity compared to controls. Using a support vector machine algorithm, RSFC features demonstrated good performance in classifying the HIE and healthy newborns in separate groups. Our results indicate the utility of fNIRS-connectivity patterns as potential biomarkers for HIE and fNIRS as a new bedside tool for newborns with HIE.

## Introduction

Hypoxic-ischemic encephalopathy (HIE) results from insufficient cerebral oxygen delivery and disrupted blood supply during the prenatal, intrapartum, or postnatal periods^1^. HIE occurs in 1 to 2 per 1000 live births in developed countries^2^. HIE is one of the most serious birth complications affecting term-born neonates and a major cause of death and long-term disabilities, including cerebral palsy, epilepsy and impairments of various cognitive functions^3,4^. Neonates with HIE are typically identified shortly after birth and deteriorate within the first days of life^1^. Therefore, early diagnosis and monitoring are key for therapeutic hypothermia (TH), which is the standard treatment for moderate to severe HIE^5^.

High spatial resolution, magnetic resonance imaging (MRI) is widely considered a gold standard for diagnosing and characterizing neurological disorders and brain injuries, including HIE^6^. However, MRI is limited by its accessibility within the neonatal intensive care unit (NICU) and sensitivity to movements, requiring imaging preferably when neonates are either asleep or sedated. Cerebral monitoring is also commonly practiced, especially during TH. Electroencephalography (EEG) based modalities are used for detecting seizures; however, they may not provide all the necessary physiological information for a continuous assessment, considering that HIE injuries are closely related to cerebral oxygenation and oxidative metabolism^1,7^. NIRS has been used to examine cerebral hemodynamics and oxygenation changes during the pre-TH period, as well as during and after rewarming^8^. In the first 6 h of life following injury due to hypoxia–ischemia (HI), cerebral oxygenation decreases but recovers by 18–20 h of life. Cerebral oxygenation changes are predictive of brain injury in this population^9^. Increased cerebral oxygenation during 24–36 h of life significantly increased the likelihood of developing brain injury^10^. Increases in cerebral oxygenation may reflect mitochondrial changes due to decreased oxygen utilization. Cerebral perfusion may exceed metabolism demands reflected in increased cerebral oxygenation.

Resting-state functional connectivity (RSFC) offers new perspectives for examining early brain injuries. RSFC, typically derived from functional MRI (fMRI) images, explores the functional synchronization among brain regions and reveals the organization of brain networks, for adults and infants^11–14^. RSFC has been widely introduced to study prematurity and early brain injuries^15^. Chiarelli et al. used multivariate analysis to find spatially diffused alterations of RSFC in neonatal cortices^16^. In preterm-born neonates with post-hemorrhagic infarction, reduced functional connectivity (FC) was reported^17^. In another cohort of preterm-born neonates with periventricular hemorrhage, reduced connectivity within the motor networks was also reported^18^. In newborns with HIE, functional networks have been explored with reported reduced inter- and intra-hemispheric connectivity strength^19^, particularly in primary motor regions^20^. Also, functional connectivity and graph-theory measurements were positively associated with developmental outcomes^21^.

Functional near-infrared spectroscopy (fNIRS), similar to fMRI, can also yield cerebral RSFC for neonates. However, fNIRS is more accessible for the NICU population and insensitive to head motion. fNIRS measures the amount of near-infrared light absorbed by hemoglobin, which is converted into concentration changes of oxygenated and deoxygenated hemoglobin (HbO and Hbr), respectively as an indirect measurement of neural activity^22^. RSFC derived from fNIRS and fMRI is comparable in several studies on adults^23^ and a 1 month-old infant^24^. Less is known about the comparability between fNIRS and fMRI in the newborn period. Considering the rapid growth and maturation of the brain during the early months of life^25^, the penetration depth of fNIRS can vary (i.e., portions of white and grey matter covered by near-infrared light can be different during early development of the brain). As the key modulator of penetration depth, an optimal source-detector distance for infants is still being discussed^26^. Since fMRI RSFC is largely based on grey matter, RSFC of fNIRS may differ from fMRI in newborns.

fNIRS RSFC has also been examined in previous studies with critically-ill neonates to determine whether this analytic method can be used as a biomarker for brain health. In preterm-born neonates, altered FC was reported at term equivalent age compared to term-born neonates^27^. Lower FC on short-range and inter-hemispheric connections has also been reported in the preterm population^28^, associated with brain injury and larger ventricular volumes^29^. Few studies to date have examined RSFC using fNIRS to examine the impact of brain injuries after term birth. Zhang et al.^30^ reported, in a small sample of newborns with HIE (n = 13), who were examined with fNIRS in the pre-TH period, that decreased RSFC patterns compared to a comparable number of healthy newborns. Reduced long-range connectivity was reported in the HIE cohort. Graph theory measures applied to the data indicated that newborns with HIE had In increased ability for loIal information transmission, reflected in higher local network efficiency. The findings could reflect compensation mechanisms. Further, graph theory-based measures such as small-worldness effectively distinguish HIE and healthy newborns. This study provided key evidence for altered FC at baseline before TH was initiated in the HIE population.

The short-term effects of the injury associated with HI in newborns remain poorly understood. fNIRS-based RSFC is a promising avenue for biomarker identification in this vulnerable population. In the current work, we examined fNIRS-based RSFC in newborns with HIE, of whom the majority were assessed post-TH after rewarming. Our central hypothesis was that fNIRS-based RSFC would be decreased in newborns with HIE and that graph theory-based measures of these metrics would be viable metrics for classifying these newborns. Our first aim was to compare patterns of RSFC obtained using fNIRS and fMRI to determine whether similar findings would be obtained between the two modalities. As part of a sub-aim, we further examined FC obtained using both fNIRS and fMRI in relation to structural MRI-based volumes of grey and white matter as additional markers of brain health. The second aim of our study was to compare fNIRS-based RSFC between term-born neonates with HIE and healthy controls. We subsequently examined whether graph theory-based metrics would serve as useful indicators of an HIE diagnosis. Using a support vector machine model applied to the HIE and healthy newborn data, we determined whether graph theory measures would predict diagnostic group membership.

## Methods

### Participants

This study was conducted as part of an ongoing study investigating brain injury in critically-ill neonates. Participants were recruited between April 2021 and August 2022. Participants with HIE were recruited from the NICU at the Children’s Hospital of South Western Ontario, London, Canada. Eligibility criteria for the patients with HIE included: HIE diagnosis, gestational age (GA) ≥ 36 weeks, birth weight ≥ 2000 g. HIE diagnosis was based on a cord gas pH of ≤ 7.0 and/or base deficit of ≥ 16 mmol per L; if pH was between 7.01 and 7.15 or a base deficit was between 10 and 15.9 mmol per L, additional history of an acute perinatal event and an APGAR score at 10 min of ≤ 5, or need for assisted ventilation/resuscitation at birth, and the presence of seizures or evidence of moderate or severe encephalopathy based on a standardized neurological examination^31^. We excluded neonates if they had evidence of major anomalies of the brain or other organs, congenital infections (e.g., TORCH), intrauterine growth restriction (IUGR), identifiable metabolic disorder or ultrasound evidence of a large parenchymal hemorrhagic infarction.

Term-born healthy newborns with no reported brain injury were also recruited to the study from the Mother Baby Care Unit (MBCU). Inclusion criteria were admission to the MBCU, birth > 36 weeks’ GA, and inborn. Exclusion criteria were evidence of congenital malformation or syndrome, antenatal infections, antenatal exposure to illicit drugs, small for gestational age (SGA) and IUGR.

The study was approved by the Health Sciences Research Ethics Board at Western University. Informed consent was provided by the parents/caregivers of the newborns. The study was conducted in accordance with the Declaration of Helsinki.

### Clinical and demographic variables

Maternal and newborn health data were extracted from electronic medical records by a Paediatric Nurse, Paediatric Resident or NICU Fellow. For participants with HIE, the demographic data extracted included gestational age, birth weight, biological sex, HIE stage (based on Sarnat staging^32^), resuscitation details, Apgar scores and cord pH. We also collected the following data: 72 h treatment with TH, the presence of brain injury on MRI, and postnatal infections (clinical sepsis or positive culture infection, confirmed necrotizing enterocolitis).

### MRI image acquisition

Newborns with HIE underwent at least one MRI scan post-TH after rewarming. Healthy newborns did not undergo MRI scanning. Newborns with HIE were scanned on a 1.5 T GE MRI scanner. A T1-weighted structural image was acquired (TR = 8.4–11.5 ms [depending on clinical requirements], TE = 4.2 ms, flip angle = 12/25°, matrix size 512 × 512, 99–268 slices, voxel size typically 0.39 × 0.39 × 0.5 mm (0.31 × 31 × 5–0.43 × 0.43 × 0.6 for some neonates), as well as a T2-weighted structural image (TR = 3517–9832 ms, TE = 7.3–8.4 ms, flip angle = 90/160°, matrix size 256 × 256, 19–60 slices, 0.7 × 0.7 × 2–5 mm voxel resolution). Additionally, an echo planar imaging sequence to measure Blood Oxygen Level–Dependent (BOLD) fMRI data was also acquired to examine RSFC (TR = 3000 ms, TE = 50 ms, flip angle = 70°, matrix size 64 × 64, 39 slices, voxel size 3 × 3 × 3 mm, total volumes 35).

### fMRI preprocessing and analysis

**Figure 1:**
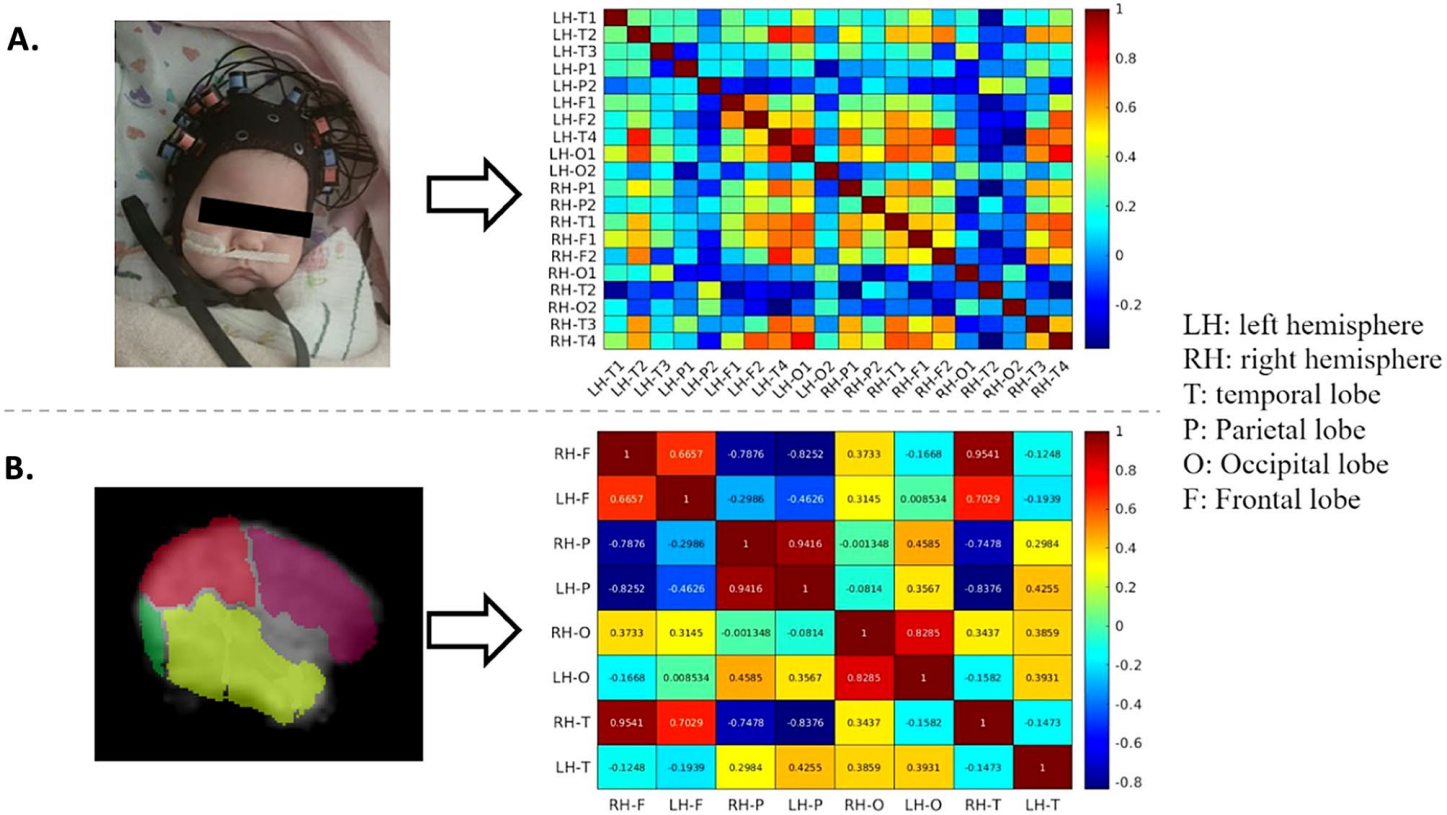
(**A**) Newborn wearing the fNIRS cap (family provided informed consent for publication of the image). Based on fNIRS recordings of oxygenated hemoglobin (HbO), 20-by-20 connectivity maps were created. (**B**) Atlas (lobe)-based analysis of the fMRI data. A hemisphere is sectioned into temporal, parietal, frontal and occipital lobes. 8-by-8 connectivity maps were built upon fMRI data.

The fMRI data were preprocessed using FMRIB Software Library^33^ (https://fsl.fmrib.ox.ac.uk/fsl/fslwiki/FSL). The preprocessing pipeline included brain extraction, motion correction, spatial smoothing (full width at half maximum = 5 mm), band-pass filtering (0.01–0.2 Hz) and registration to a neonatal atlas^34^. From the frontal, parietal, temporal and occipital lobes of both hemispheres, we extracted the average BOLD sequences. Then, we calculated the Pearson correlation among them to build an 8-by-8 RSFC map for each neonate (Fig. 1B).

### T1-weighted image processing and segmentation

The T1-weighted scans were automatically segmented using infant FreeSurfer^35^. Automatic processing steps included intensity normalization, skull stripping, and segmentation of the cortex, white matter and subcortical structures^35^. A multi-atlas approach was employed for the segmentation. Multiple brain atlases of newborns were first registered to native space, and structure labels were transferred. The atlases were developed from neonatal MRI scans^36^. To initially create the atlases, manually segmented labels were developed using MRI scans from a representative sample of neonates (0–2 years of age). In the current study, developmentally appropriate atlases for newborns were employed. Labels were fused into a single segmentation result, providing higher accuracy than single-atlas approaches^37^. Volumetric measurements for anatomical features could then be extracted^35^. Brain subcortical brain structure volumes were extracted, as well as cerebral white matter and cerebral cortex volumes, to compute total cerebral volumes (TCV). Each segmented T1-weighted image was visually inspected using Freeview software, available within FreeSurfer. Manual segmentation was employed to correct any segmentation errors (i.e., partial volume effects) using ITK-SNAP (http://www.itksnap.org/).

### Brain injury characterization

A pediatric neuroradiology fellow (LTARM) scored the T1-weighted anatomical images for brain injury severity. White matter injury (WMI) was defined as foci exhibiting T1 hyperintensity without T2 hypointensity or by low-intensity T1 foci and was scored on a 3-point scale (none = 0, minimal = 1, moderate-severe = 2–3 combined) using the methods of de Vries^38^. Intraventricular haemorrhage (IVH) was graded (none = 0, mild = 1–2, and moderate-severe = 3–4) using Papile’s method^39^. Only supratentorial injuries were scored.

### fNIRS acquisition and analysis

fNIRS was acquired in newborns with HIE (within 3 days post-TH after rewarming) and healthy newborns using the same experimental setup. Participants were scanned using a NIRSport2 (NIRx, Berlin, Germany) system with 8 light sources and 8 detectors covering the whole brain. The system operates at two wavelengths of light (760 nm and 850 nm), and the sampling rate was 10.1725 Hz. For either hemisphere, 4 channels were located on the temporal, 2 on the parietal, frontal and occipital lobes (Fig. 1A). fNIRS scans lasted for a minimum of 6 min during rest or natural sleep with the neonates lying in the incubator, cot, or caregiver’s arms.

After data acquisition, Homer3 software^40^ was used for preprocessing. The pipeline included spline interpolation for motion correction^41^, band-pass filtering of 0.01–0.1 Hz and conversion to HbO and Hbr fluctuations with modified Beer-Lambert law^42^. For each neonate, two connectivity maps, sized differently, were calculated using Pearson correlation. Channels corresponding to one lobe were averaged and then correlated with other cortices to build lobe-wise connectivity maps (8 by 8). 20-by-20 channel-wise RSFC maps were also calculated with individual channels corresponding with each other. HbO and Hbr data were both used for building connectivity maps separately (Fig. 1A).

### Statistical analysis

Statistical analyses were performed using Matlab (R2020b, Natick, Massachusetts: The MathWorks Inc).

For the first aim, which was comparing the RSFC maps yielded from the fNIRS and fMRI data, Euclidean distances and Jaccard distances were calculated between the two 8-by-8 maps at various levels of sparsity^43^. It is considered common practice to filter out negative or low-weighted connections when analyzing RSFC maps, where the percentage of connections remaining is the level of sparsity of a map. For weighted maps, we kept the original weights of connections, while for binarized maps, the remaining connections after filtering were rounded to 1. Given adjacency matrices of two maps ${A}_{1}=\left[{a}_{ij}^{1}\right]$ and ${A}_{2}=\left[{a}_{ij}^{2}\right]$, where ${a}_{ij}$ was the weight of connection between node $i$ and $j$, Euclidean distance between the two was defined as

1 $${d}_{E}=\sqrt{\sum_{{i,j}}{\left({a}_{ij}^{1}-{a}_{ij}^{2}\right)}^{2},}$$

while Jaccard distance was defined as

2 $${d_J} = 1 - J,\quad {\text{where}}\quad J = \left\{ {\begin{array}{*{20}{l}} {\frac{{\mathop \sum \nolimits_{i,j} \min \left( {a_{ij}^1,a_{ij}^2} \right)}}{{\mathop \sum \nolimits_{i,j} \max \left( {a_{ij}^1,a_{ij}^2} \right)}},}&{\;if\;\mathop \sum \limits_{i,j} \max \left( {a_{ij}^1,a_{ij}^2} \right) > 0} \\ {1,}&{\;otherwise} \end{array}} \right.$$

We also calculated similarity maps for weighted and binarized RSFC maps between fNIRS and fMRI. Note that similarity maps had the same dimension as connectivity maps. For weighted cases, entries of similarity maps were the average Euclidean distance among subjects for the corresponding connections. In contrast, for binarized cases, they were the percentage of subjects sharing the connection. Volume counts of cortical grey matter obtained from T1-weighted images were associated with overall fNIRS and fMRI connectivity strength, respectively, using linear regression, adjusted for GA at birth, postmenstrual age (PMA) at scan and sex.

To address the second aim, which was identifying altered connectivity patterns between HIE neonates and healthy controls, we used graph theory-based measurements to quantify the patterns of 20-by-20 RSFC maps. On HbO and Hbr maps, we calculated the clustering coefficient of a map as

3 $${C}_{p}=\frac{1}{N}\sum_{i}\frac{\sum_{j,k}{a}_{ij}{a}_{ik}{a}_{jk}}{\left(\sum_{j}{a}_{ij}-1\right)\sum_{j}{a}_{ij}},$$

where $N$ was the number of nodes in the map. Network efficiency was also calculated locally as

4 $$NE=\frac{1}{N}\sum_{i}\left[\frac{1}{\left|{G}_{i}\right|\left(\left|{G}_{i}\right|-1\right)}\sum_{k\ne j\in {G}_{i}}\frac{1}{{d}_{jk}}\right],$$

where ${d}_{ij}$ was the shortest distance ${N}_{i}$ between $i$ and $j$, ${G}_{i}$ the node set containing $i$ and its direct neighbors only, and $\left|{G}_{i}\right|$ the number of nodes of ${G}_{i}$. Modularity was defined as

5 $$M=\sum_{p}\left[{e}_{pp}-{\left(\sum_{q}{e}_{pq}\right)}^{2}\right],$$

where $C$ was the number of communities identified by a Louvain-like algorithm^44^ and ${e}_{pq}$ denoted the fraction of connections between community $p$ and $q$. Based on clustering, the between-subject variation was calculated using mutual information^45^. Given a confusion matrix $C=\left[{c}_{wv}\right]$, where ${c}_{wv}$ was the number of nodes in both community $w$ and community $v$ of two separate clusterings, respectively, mutual information was defined as

6 $$MI=\frac{-2\sum_{w,v}({c}_{wv}{\text{ln}}\frac{{c}_{wv}N}{\sum_{w}{c}_{wv}\sum_{v}{c}_{wv}}) }{\sum_{w}\left(\sum_{v}{c}_{wv}{\text{ln}}\frac{\sum_{v}{c}_{wv}}{N}\right)+\sum_{v}\left(\sum_{w}{c}_{wv}{\text{ln}}\frac{\sum_{w}{c}_{wv}}{N}\right)}.$$

T-tests were used to determine if the two groups were significantly different between a measurement.

To further demonstrate the distinctive power of connectivity measurements separating HIE neonates from healthy controls, we trained machine learning models of support vector machine (SVM) with several local metrics, including connectivity, clustering coefficient, nodal efficiency, degree centrality and closeness centrality, with fourfold cross validation. The accuracies of classification were calculated, and receiver operating characteristic (ROC) curves were plotted. Areas under ROC curves (AUC) were also obtained.

### Consent statement

Consent was obtained from the guardians of the study participants.

## Results

### Participants

Twenty-one term-born neonates with HIE were enrolled (GA at birth = 38.92, SD = 1.55 weeks, Table 1). Nineteen (90.4%) of the HIE neonates underwent TH before they were scanned with MRI and fNIRS. Two newborns who did not undergo TH were diagnosed with mild or moderate HIE. All newborns with HIE underwent an fNIRS scan at a PMA of 39.96, SD =  1.43 weeks after rewarming. Twelve (57%) of the HIE neonates had MRI scans (PMA = 39.94, SD =  1.15 weeks).

**Table 1:** Participant characteristics.

	HIE participants N = 21	Healthy newborns N = 20	P-value
Gestational Age, *weeks [SD]*	38.92 [1.55]	39.08 [1.21]	0.72
Sex, *male n, (%)*	12 (57.14)	9 (45.00)	0.4
Birth Weight, *kgs [SD]*	3.13 [0.59]	3.49 [0.42]	0.08
PMA at scan, *weeks [SD]*	39.96 [1.43]	39.93 [1.27]	0.95

The median values and interquartile ranges. Probability values provide results using the t-test for continuous measures and Chi-square tests for categorical measures. PMA: postmenstrual age.

Twenty healthy term-born infants were recruited from the MBCU of LHSC with a birth GA of 39.08, SD =  1.21 weeks, and fNIRS scans were performed within 48 h of life (Table 2). None of the healthy newborns underwent an MRI.

**Table 2:** Characteristics for HIE participants.

	HIE newborns (*n* = *21*)
First pH (cord or peripheral)	Mean = 6.98, SD = 0.16
	Min = 6.8, Max = 7.4
	25th Percentile: 6.90
	50th percentile: 6.92
	75th percentile: up to 7.03
Apgar 5 min	Mean = 3.94, SD = 2.6
	Min = 0, Max = 9
	25th Percentile: 2.25
	50th percentile: 3.5
	75th percentile: 5.75
Sarnat staging	Mild: 3 (14.3%)
	Moderate: 15 (71.4%)
	Severe: 3 (14.3%)
Therapeutic hypothermia	19 (90.5%)
Intubation at birth	10 (47.6%)
Days of ventilation	Mean = 4 days, SD = 7.0
	Min = 0 days, Max = 28 days,
	SD = 5.11 days
	25th Percentile: up to 0 days
	50th percentile: up to 1.0 days
	75th percentile: up to 5.3 days

### Brain injury characterization

Most newborns with HIE (n = 20, 95%) had white matter injury identified using MRI (Table 3). Mild white matter injury was commonly seen in the neonates, regardless of HIE severity (n = 19, 90%). Injury to the basal ganglia was identified in one (5%) neonate with severe HIE. The presence of IVH was only seen in one (4%) participant with severe HIE. No watershed injury was evident in any MRI scans of the newborns with HIE.

**Table 3:** Brain injury patterns in HIE participants.

HIE severity	Grade	Mild (n = 3)	Moderate (n = 15)	Severe (n = 3)
*White matter injury, n [%]*	Grade 1	3[67]	14[93]	2[67]
	Grade 2	0	0	1[33]
	Grade 3	0	0	0
*Intraventricular haemorrhage, n [%]*	Grade 1	0[0]	0[0]	0[0]
	Grade 2	0[0]	0[0]	1[33]
	Grade 3	0[0]	0[0]	0[0]
*Basal ganglia, n [%]*		0[0]	0[0]	1[33]
*Watershed injury, n [%]*		0[0]	0[0]	0[0]

White matter injury was scored according to de Vries^38^. Intraventricular haemorrhage grade was using scored using the method of Papile^39^; WMI using the method of de Vries; Positive = any morphological abnormality.

### Comparing fNIRS and fMRI

**Figure 2:**
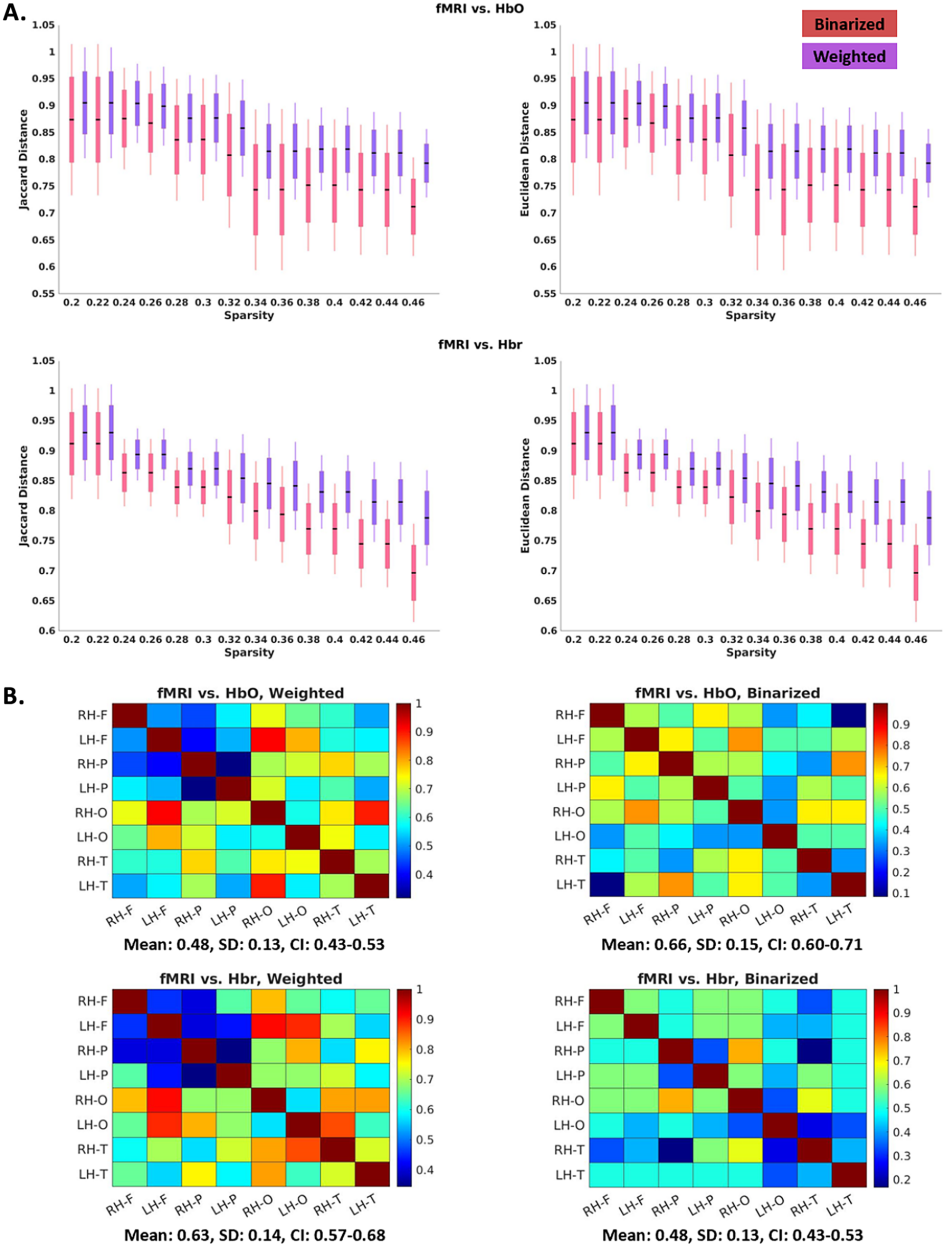
(**A**) Similarity of RSFC maps between fMRI with respect to HbO and Hbr, measured by Euclidean distance (right panel) and Jaccard distance (left panel). Both weighted and binarized maps were implemented. (**B**) Lobe-wise similarity maps at a sparsity of 45%. Color bar denotes the value of similarity. Higher values reflect greater similarity between the two modalities.

Lobe-wise 8-by-8 connectivity maps were obtained from fNIRS for HbO and Hbr for neonates with HIE. Euclidean and Jaccard distances were used to compare fNIRS and fMRI connectivity maps at levels of sparsity (Fig. 2A). Means, standard deviations (SD) and 95% confidence intervals (CI) of the two metrics were calculated based on subject-by-subject comparisons. Sparsity ranged from 0.2 to 0.46. We also calculated the similarity map (Fig. 2B) between the two modalities.

**Figure 3:**
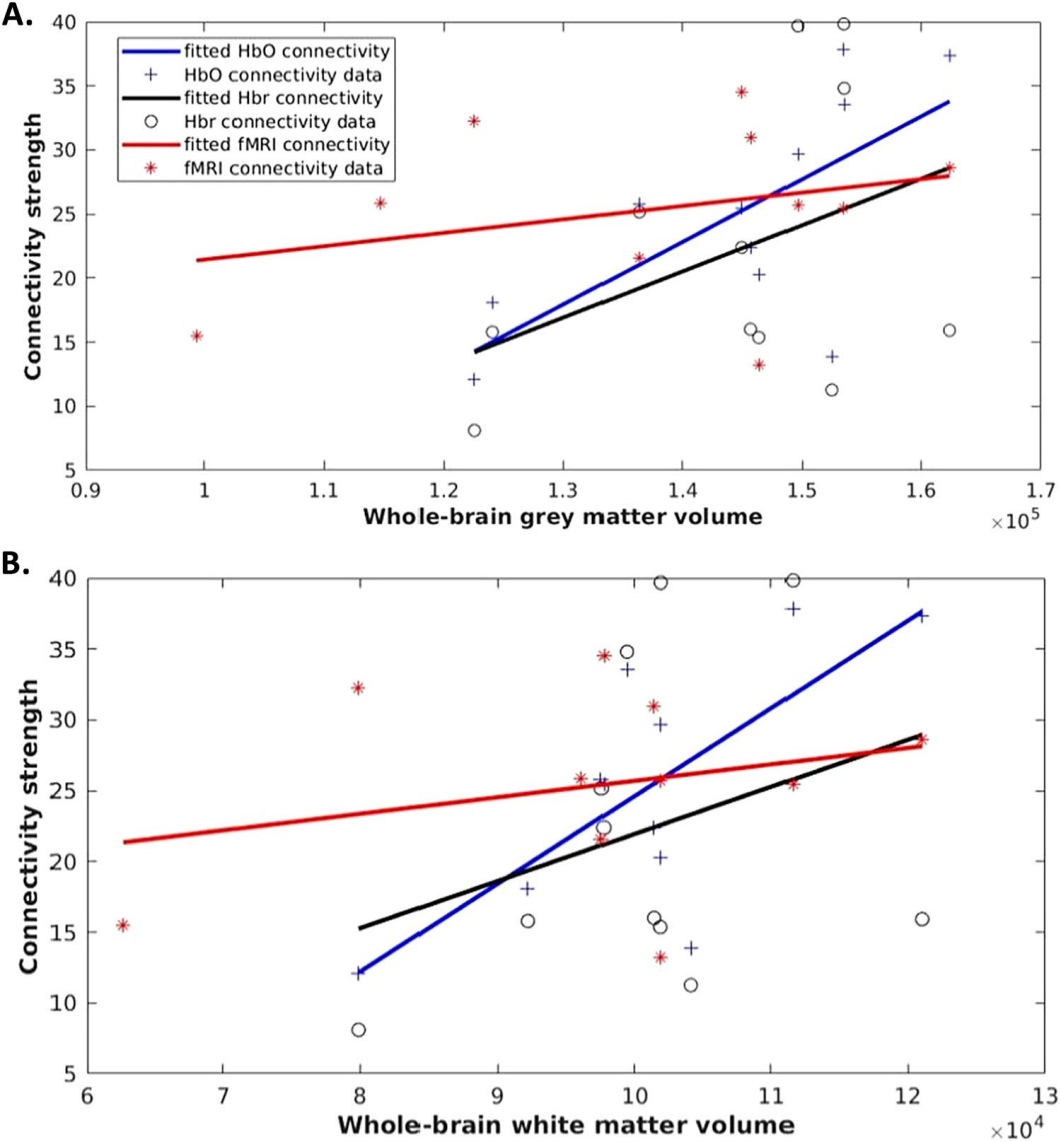
Brain volumes and fNIRS- and fMRI-based functional connectivity strength. Functional connectivity strength (y axis) of oxygenated (HbO, blue lines and plus [+] signs), deoxygenated (HBr, black lines and open circles [o]) hemoglobin and fMRI-based functional connectivity (red lines and stars [*]) in relation to grey matter volumes (**A**, x axis) and white matter volumes (**B**, x axis).

We also examined the association between brain volume and FC (Fig. 3). Correlations were calculated with whole-brain grey matter or white matter volume against HbO, Hbr or fMRI connectivity. Connectivities were summed over all connections, which were weighted and not filtered. We showed that FC increases with either grey or white matter volume. HbO connectivity showed a significant positive correlation with grey and white matter volumes (p-values were 0.015 and 0.011, respectively), adjusted for birth GA, PMA at scan and sex. While Hbr and fMRI connectivities were not significantly correlated with volumes, they demonstrated slight positive increases in relation to the brain volumes.

### Altered RSFC patterns of neonates with HIE

**Figure 4:**
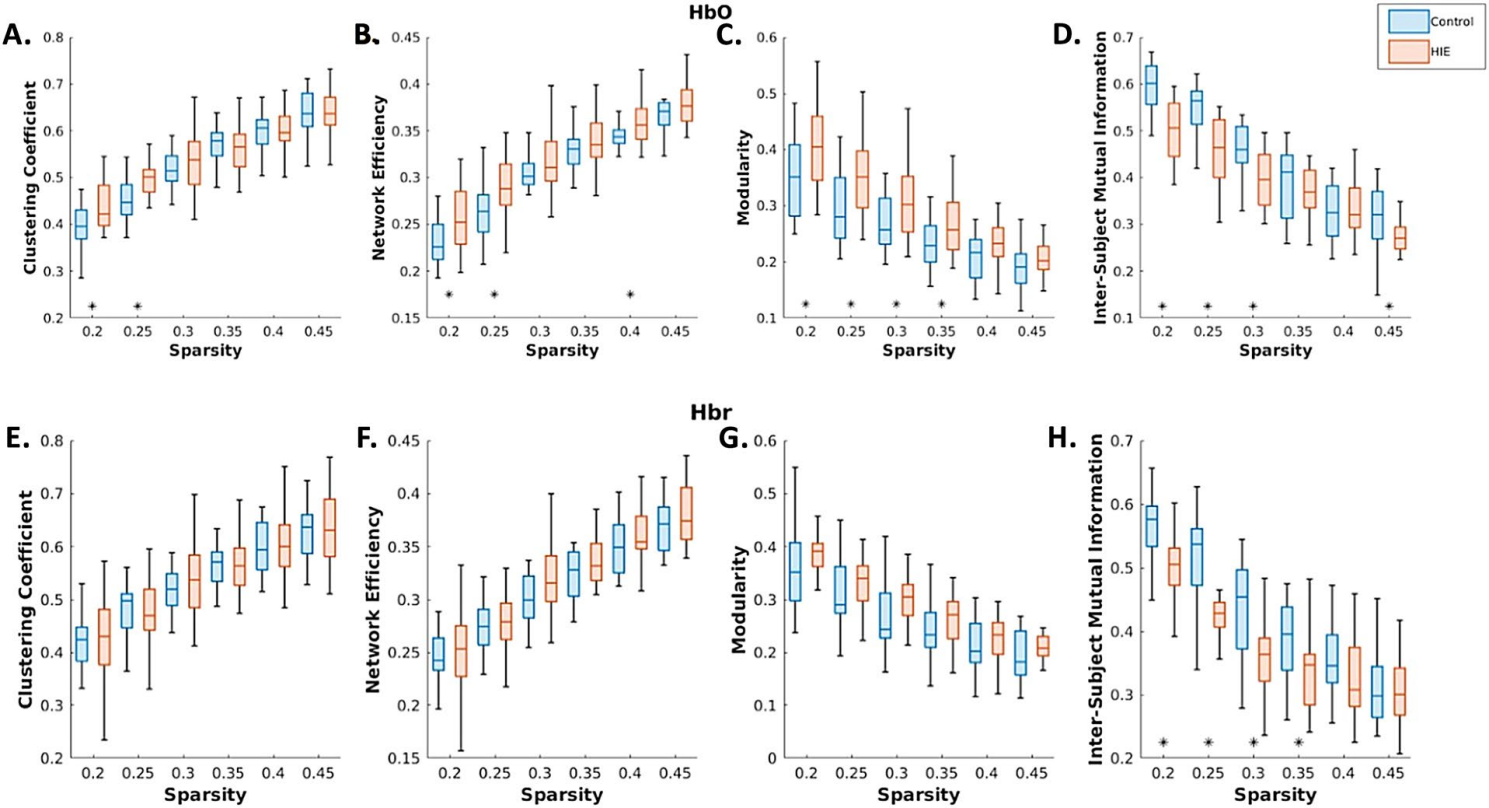
Graph theory-based metrics measuring the differences in fNIRS RSFC patterns between newborns with HIE (red) and healthy controls (blue). The metrics were calculated at various levels of sparsity (0.2–0.45). *p < 0.05.

Based on the connectivity maps obtained from fNIRS data, graph theory-based metrics were calculated to characterize patterns of RSFC and identify differences between HIE and control groups (Fig. 4). It was shown that the HIE group had increased clustering coefficient, network efficiency and modularity compared to controls, but had lower consistency within the group.

**Figure 5:**
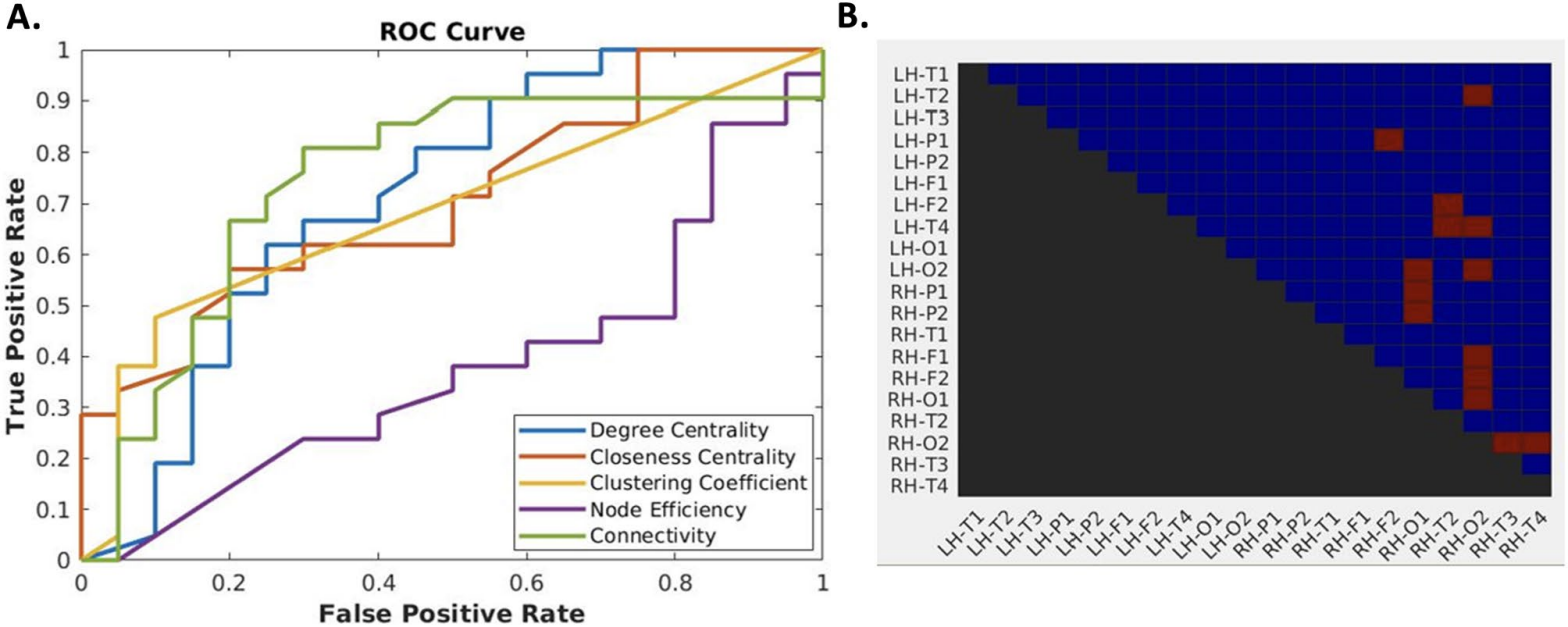
(**A**) ROC curves of SVM classifiers trained with connectivity features. In the order shown in the legend, maximal accuracies of individual feature sets were 68.29%, 68.29%, 68.29%, 51.22% and 75.61%, respectively. AUCs were 0.71, 0.71, 0.69, 0.36 and 0.75, respectively. (**B**) The connections marked red were those showing significance based on t-tests and were selected to train the SVM model.

Local measurements, including connectivity, degree centrality, closeness centrality, clustering coefficient and node efficiency, were calculated and used as features for training SVM classifiers to distinguish the HIE group from the control. Note that only features showing significance in t-tests were selected to train SVM models. We achieved a maximal accuracy of 75.61% and a maximal AUC of 0.75 with connectivity features (Fig. 5A) using fourfold cross validation.

## Discussion

Despite the wide adoption of TH along with other interventions, HIE remains a major cause of mortality as well as later physical and cognitive difficulties. In this study, we examined functional connectivity patterns in a heterogeneous cohort of newborns with HIE using multi-modal imaging methods. RSFC maps yielded from fNIRS and fMRI were comparable within the cohort of HIE newborns. Further, positive associations between grey and white matter volumes, a marker of brain health, and fNIRS and fMRI connectivity strength were evident. Our findings provide support for fNIRS to be a promising bedside monitoring method to be used alongside other imaging technologies, especially for newborns receiving care in the NICU. We also demonstrated altered fNIRS-based connectivity patterns in HIE neonates compared to healthy newborns and the distinctive power of these connectivity features with machine learning models. Results indicated that fNIRS-based RSFC may be a novel biomarker of brain health in HIE.

The RSFC maps derived from fNIRS (HbO or Hbr) were comparable to fMRI in newborns with HIE who underwent TH. Our findings align with previous studies demonstrating good correspondence between fNIRS and fMRI. In adults, Sasai et al.^46^ reported good spatial correspondence between fNIRS and fMRI on several resting-state networks using seed-based analyses. Further, Duan et al.^23^ in an adult population reported positive correlations between fNIRS and fMRI connectivity strength as well as similar topological patterns at different levels of sparsity. Anwar et al.^47^ recorded simultaneous fNIRS, fMRI and EEG in adults to show bi-directional connectivity that occurs within sensorimotor networks, using Granger causality. In infants, less work has focused on comparing resting-state networks using fNIRS and fMRI. However, Bulgarelli et al.^24^ demonstrated a strong resemblance between fNIRS and fMRI connectivity using dynamic causal modeling in a 6-month-old infant.

In the current work, we further examined fNIRS- and fMRI-based connectivity in relation to grey and white matter volumes as separate markers of brain health. Previous MRI studies conducted during the early stages of brain development demonstrated positive associations between FC strength and brain volumes^48–50^. fNIRS-based connectivity was significantly associated with both grey and white matter volumes. Our findings indicate that in the hypoxic newborn, FC can be maintained while adapting to changes in brain volume. However, this finding was primarily driven by HbO-based connectivity. In contrast, fMRI- and Hbr-based connectivity only showed positive trends towards an association with brain volumes. The physiological basis of the BOLD and Hbr signals are comparable in that they are both sensitive to changes in Hbr and are coupled to underlying changes in neuronal activity. In turn, connectivity based on deoxygenation changes may show less association with grey and white matter development in the rapidly changing newborn brain, as these processes are supported by synaptogenesis and myelination and are highly dependent on oxygen and nutrient delivery. An additional consideration is that the temporal dynamics of RSFC have been widely documented^51,52^, and methodological differences in the fNIRS and fMRI acquisition and analyses could further explain the inconsistency in the results.

When comparing fNIRS-based RSFC between neonates with HIE and healthy controls, the HIE group showed increased clustering coefficients, network efficiency and modularity. These metrics are commonly considered to be indicative of the small-worldness of networks. Small-worldness is characterized by high clustering and short path length and is commonly seen in brain networks^53,54^. Findings suggest that brain regions with similar functions demonstrated more synchronized activity and had more highly connected hubs that support efficient global communication. Similar findings were documented by Gozdas et al.^55^, who reported increased modularity in preterm-born neonates, but using fMRI-based connectivity metrics. Additionally, using fNIRS, Zhang et al.^30^ also reported increases in several small-world metrics in newborns with HIE. An increase in small-worldness in the HIE population may reflect disruptions in the underlying white matter fibre pathways that may be reflected in severed long-range connectivity but strengthened local connectivity through neural compensation and reorganization. These compensatory mechanisms may be a common feature in neonates born critically ill^56^ that can even be seen later in adulthood^52^ or neurological disease states^53^. Increased functional connectivity within local networks has been reported previously. For example, newborns scanned with fNIRS demonstrated positive associations between connectivity strength of homologous-interhemispheric networks as well as behavioural measures of negative emotionality^57^. In relation to the current study, our findings of increased functional connectivity could also partially be influenced by exposure to invasive NICU procedures (TH, tape removal, skin breaks, etc.) experienced by the newborns. Further, increased grey matter cerebral blood flow within sensorimotor networks has been reported in preterm born infants^58^, which may have also been a contributing factor to the increased functional connectivity within the networks seen in our study. Future studies could address whether these changes are related to underlying brain injuries associated with HI and predict local changes in connectivity. Further, an increased variation of clustering among neonates with HIE was evident and could also be a result of variations in the severity of HIE, IVH, or be related to white matter injury or a combination of these factors. Given our sample size, we could not further assess whether the variations in clustering were related to clinical factors or variations in care practices, but this could be explored in future work.

Machine learning models were implemented in the current study to determine whether RSFC features can be potential biomarkers of HIE. Optimal performance was realized when using connectivity features that were a product of significant connections that were either interhemispheric or within the right hemisphere. Altered interhemispheric or long-range connections have been reported in multiple studies^19,20^, which may be related to HIE-induced injury or inflammation localized to the white matter or even subcortical areas, which are key to long-range neuronal signal transmission. Connections within hemispheres were significantly different in the right but not the left hemispheres. Further examination as to whether there may be hemispheric vulnerability to HIE is needed. Massaro et al.^54^ demonstrated that the magnitude of cerebral pressure in the right hemisphere was predictive of adverse outcomes detectable by MRI. Whether a similar lateralization effect due to changes in intracranial pressure occurred in the current sample remains unknown but could have been an underlying factor.

The majority of the HIE participants were scanned after TH and rewarming. The two participants who had mild HIE were not candidates for TH. Given that these newborns represented a small fraction of the total dataset, their data were unlikely to have unduly influenced the results. While TH is the standard of care for newborns with severe or moderate HIE, our findings demonstrating altered RSFC maps suggest that disrupted functional organization of the brains persists after TH. The persisting disrupted RSFC patterns could also contribute to adverse developmental outcomes in the first few years of life^55,56^. The present study focused on RSFC patterns in newborns with HIE, and future studies should incorporate long-term developmental outcomes. This would be the next step to determine how fNIRS markers of brain health at the bedside can predict developmental abilities in newborns with HIE.

The strengths of our study are grounded in several areas. Firstly, this was a single-centre study with a well-characterized cohort of newborns with HIE and healthy newborns. Also, a high-density fNIRS system offered coverage of the newborn brain's frontal, parietal, temporal and occipital areas. We also had the opportunity to characterize functional networks that were related to brain structure in the HIE population; however, not all participants had the same measures available. The MRI scans were conducted as part of clinical care and after TH and rewarming. Given the timing of the MRI scans, some families could only consent to the fNIRS component of the study. While the goal of the study was to compare RSFC maps obtained using fNIRS and fMRI, these two modalities could not be obtained at the same time in our sample due to a lack of resources in a clinical imaging centre. The duration of the fMRI scanning sequence was relatively short. However, the neonates underwent clinical imaging before the research sequences and could only be away from the NICU for a short period of time. The fMRI protocol was tested in an adult prior to scanning the newborns, further the networks were assessed in individual newborns to determine their robustness. Additionally, the fNIRS probes were not precisely located in the MRI space, and the fMRI data were analyzed using an atlas approach. In turn, the spatial correspondence between the fNIRS and fMRI data could be improved in future work. Another consideration is that the fNIRS data may have been influenced by physiological noise. In adults, short-distance channels are frequently implemented in fNIRS studies as a means to assess surface-artefacts, signal quality, source localization, and improve signal decomposition^59^. However, in neonates placing short distance channels on channels that already very close together is ineffective due to the smaller surface area of the head in neonates compared to adults^60^. Another approach for future studies with neonates would be to collect physiological measurements (i.e., heart rate) using external monitoring and use these measurements to improve signal decomposition.

## Conclusions

In this study, we determined the utility of fNIRS to assess RSFC as a potential biomarker for brain health in term-born neonates with HIE. By incorporating a multimodal neuroimaging investigation using fNIRS, fMRI and structural MRI, we showed that fNIRS-based RSFC patterns were comparable to those seen with fMRI and that these metrics were predictive of brain volumes. We further demonstrated that fNIRS-based RSFC maps differed between HIE and healthy newborns. Lastly, fNIRS-based connectivity metrics performed well in machine learning models to determine diagnostic group membership. Overall, study findings indicate that altered fNIRS-based RSFC patterns can be potential biomarkers of HIE. fNIRS at the bedside could be complementary to other imaging modalities in terms of characterizing neonatal brain function.

## Data Availability

The datasets generated and/or analysed during the current study are available upon reasonable request.
